# Multiple drug allergies in a patient with acute pancreatitis: case report

**DOI:** 10.3389/fmed.2025.1564218

**Published:** 2025-05-09

**Authors:** Qin Shang, Yuanxi Jiang, Zhengyi Yang, Diao Yu, Li Qin, Xu Zhang

**Affiliations:** ^1^Department of Science Research, Zhejiang Provincial People’s Hospital Bijie Hospital, Bijie, China; ^2^Department of Gastroenterology, Zhejiang Provincial People’s Hospital Bijie Hospital, Bijie, China; ^3^Department of Medical Laboratory, Zhejiang Provincial People’s Hospital Bijie Hospital, Bijie, China

**Keywords:** multi-drug allergy, acute pancreatitis, ceftriaxone, gabexate mesylate, case report

## Abstract

This study mainly reports a case of a middle-aged man who was admitted to the hospital due to sudden upper abdominal pain after several hours. After being given the painkiller compound diclofenac sodium in the outpatient clinic, the patient suffered from systemic itching, redness, and rash. Acute pancreatitis was diagnosed after admission. During the treatment, the patient had allergic reactions to multiple drugs. This case is rare. In this case, case reports are used for improving the understanding of multi-drug allergies by clinical pharmacists and clinicians, which offers certain reference for dealing with the safety and effectiveness of medication in patients undergoing multi-drug allergies.

## 1 Introduction

Acute pancreatitis frequently occurs in adults. It is an inflammatory disease of the pancreas tissue’s own digestive system caused by abnormal activation of pancreatic enzymes. It is the main reason for admission to hospital because of gastrointestinal diseases in most patients and may result in multiple organ dysfunction (1). Medical treatment for acute pancreatitis includes supplementation of large amounts of fluids, proton pump inhibitors, somatostatin, and intravenous infusions including prophylactic use of antibiotics (2). In cases where there is a lot of pancreatic tissue necrosis, non-drug treatment can be applied, that is, minimally invasive pancreatic surgery can be performed to remove the necrotic lesion to prevent lesion expansion and exacerbation of the condition.

Multi-drug allergy refers to the patient’s allergic reaction to multiple drugs with different chemical structures at the same time (3). Drug treatment has been the most significant treatment method for the treatment of acute pancreatitis. However, some patients experience allergic reactions to multiple drugs. Therefore, discovering and solving the drug safety of this type of patient has become an urgent problem to be addressed at present. This article reports a case of acute pancreatitis who had allergic reactions to multiple drugs during admission to the hospital for medical treatment, providing certain references for clinicians.

## 2 Case report

**Figure 1:**
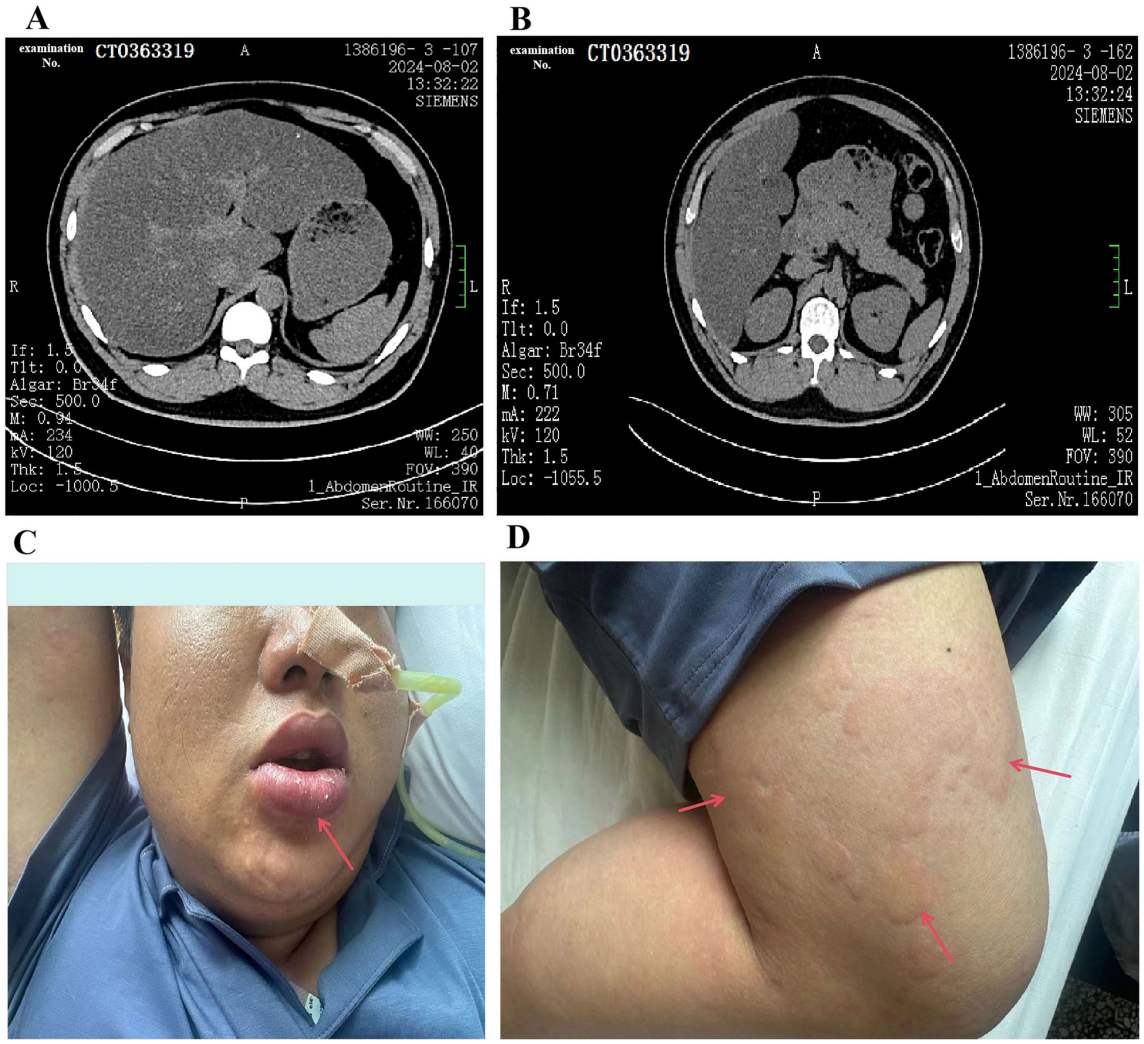
**(A)** A computerized tomography image of the patient’s liver with acute pancreatitis. **(B)** A computerized tomography image of the patient’s pancreas with acute pancreatitis. **(C,D)** Allergic reaction in a patient with acute pancreatitis after drug infusion.

The 32-year-old male patient was admitted to our hospital on August 2, 2024 due to unexplained upper abdominal pain for 5 h, accompanied by nausea and non-jet vomiting. There was no drag pain or metastatic pain. Physical examination revealed that the patient was tender throughout the body, with obvious abdominal bulge, obvious tenderness under the xiphoid process and left upper abdomen, and no percussion pain in both kidney areas. No hematemesis, melena, chest tightness, chest pain, frequent urination, urgency or disturbance of consciousness was observed. Owing to the unbearable pain, 2 mL compound diclofenac sodium (containing diclofenac sodium 25 mg and acetaminophen 150 mg) was given through intramuscular injection at the outpatient department. After a few minutes, the patient developed erythema all over the body without itching and discomfort. Meanwhile, an allergic reaction was taken into consideration. After giving 1 mL diphenhydramine hydrochloride (1 mL:20 mg) via intramuscular injection for anti-allergic treatment, the symptoms disappeared. It was initially considered that the patient was admitted to the Department of Gastroenterology of our hospital for acute pancreatitis. After admission, the blood sugar was randomly 16.67 mmol/L with the glycosylated hemoglobin being 9.6%. Considering that the patient may have diabetes, 20 IU insulin (10 mL:400 IU) was given to control blood sugar. On August 2, 2024, laboratory test results demonstrated that triglycerides (12.65 mmol/L), high-density lipoprotein cholesterol (4.39 mmol/L), C-reactive protein (12.80 mg/L), lipase (569 U/L), γ-glutamyltransferase (144 U/L), alanine aminotransferase (95 U/L), aspartate aminotransferase (55 U/L), total cholesterol (11.73 mmol/L), α-amylase (628 U/L) and glucose (16.67 mmol/L) were all elevated (Table 1). Medical imaging data and image examination results during CT upper abdominal plain scan revealed signs of fatty liver (Figure 1A) and slightly indistinct margins of the pancreatic head (Figure 1B).

**Table 1:** Multiple biochemical indicators were tested after the patient was admitted.

Inspection item	Results	Indicate	Reference range	Unit	Detection method
TP	75.8		65–85	g/L	Double urine method
ALB	44.7		40–55	g/L	Bromocresol green method
GLO	31.1		20–40	g/L	Calculation method
A/G	1.4		1.2–2.4		Calculation method
PA	245		200–430	mg/L	Immune turbidimetry
ALT	95	↑	9–50	U/L	Lactate dehydrogenase method
AST	55	↑	15–40	U/L	MDH method
AST/ALT	0.58				Calculation method
GGT	144	↑	10–60	U/L	Rate method
ALP	131	↑	45–125	U/L	NPP-substrate-AMP buffer method
TBIL	9.3		0–26	μmol/L	Diazo method
DBIL	2.0		0–4	μmol/L	Diazo method
IBIL	7.3		1.71–13.68	μmol/L	Calculation method
TBA	1.87		0–6.71	μmol/L	Enzyme cycle method
CG	0.69		0–2.7	mg/L	Latex-enhanced immunoturbidimetry
BUN	4.82		3.1–8	mmol/L	Urease-glutamate dehydrogenase method
CREA	63		57–97	μmol/L	Enzymatic
UA	402		208–428	μmol/L	Uricase-peroxidase method
eGFR-Cr	148.90			mL/min	Calculation method
TG	12.65	↑	≤1.7	mmol/L	GPO-PODmethod
CHOL	11.73	↑	0–5.18	mmol/L	Enzymatic
HDL-C	4.39	↑	1.16–1.42	mmol/L	Direct method
LDL-C	0.54	↓	2.7–3.1	mmol/L	Direct method
APO-A1	0.63	↓	1.2–1.6	g/L	Immune turbidimetry
APO-B	0.58		0–1.05	g/L	Immune turbidimetry
K	4.86		3.5–5.3	mmol/L	Indirect ion selective method
NA	132.90	↓	137–147	mmol/L	Indirect ion selective method
CL	95.13	↓	99–110	mmol/L	Indirect ion selective method
Ca	2.48		2.2–2.7	mmol/L	Azoarsenic III method
Mg	0.88		0.75–1.02	mmol/L	Xylidine blue method
p	0.89		0.85–1.51	mmol/L	Phosphomolybdate method
α-AMY	628	↑	35–135	U/L	PNP-G7 substrate method
LPS	569	↑	1–60	U/L	Methylresorufin substrate method
GLU	16.67	↑	3.9–6.1	mmol/L	hexokinase method
CRP	12.80	↑	0–6	mg/L	Immune turbidimetry

TP, Total protein; ALB, Albumin; GLO, Globulin; A/G, Albumin/Globulin; PA, Prealbumin; ALT, Alanine aminotransferase; AST, Aspartate aminotransferase; AST/ALT, Alanine/Alanine aminotransferase; GGT, γ-glutamyltransferase; ALP, Alkaline phosphatase; TBIL, Total bilirubin; DBIL, Direct bilirubin; IBIL, Indirect bilirubin; TBA, Total bile acid; CG, Cholyglycine; BUN, Urea; CREA, Creatinine; UA, Uric acid; eGFR-Cr, Estimated glomerular filtration rate-Cr; TG, Triglyceride; CHOL, Total cholesterol; HDL-C, High density lipoprotein cholesterol; LDL-C, Low-density lipoprotein cholesterol; APO-A1, Apolipoprotein-A1; APO-B, Apolipoprotein-B; K, Kalium; NA, Natrium; CL, Chlorine; Ca, Calcium; Mg, Magnesium; P, Phosphorus; α-AMY, α-amylase; LPS, Lipase; GLU, Glucose; CRP, C-reactive protein.

The above results showed that the patient had hyperlipidemia pancreatitis. Therefore, the patient was given oral bezafibrate tablets (0.4 g) three times a day to lower blood lipids. Firstly, ceftriaxone sodium for injection (2.0 g) combined with levofloxacin injection (100 mL: 0.5 g) was administered empirically once daily for anti-infective treatment. During the infusion of ceftriaxone, the patient developed mild rash and itching symptoms. The symptoms improved after the infusion of ceftriaxone was stopped. Therefore, the patient was considered to be allergic to ceftriaxone. Secondly, somatostatin (3 mg) was injected intravenously every 12 h, aiming to inhibit pancreatic secretion. Then, gabexate mesylate (0.1 g) was injected intravenously every 8 h to inhibit pancreatic enzyme activity. Next, 100 mL raw rhubarb water was injected into gastric tube three times a day for enema to relieve constipation. Local exudation was reduced by the external application of mirabilite to the abdomen. To relieve pain, phloroglucinol (80 mg) injection was given once. Symptomatic and supportive treatments including potassium supplementation and fluid replacement were used to maintain electrolyte balance. After infusion of gabexate mesylate, the patient developed systemic wheals, and rash, accompanied by itching, and swollen lips (Figures 1C,D). Gabexate mesylate was immediately stopped, and 1 mL diphenhydramine (1 mL:20 mg) was given for anti-allergic treatment. After anti-allergic treatment, the patient’s rash and itching were significantly improved. Therefore, it was considered that the patient was allergic to gabexate mesylate. Given that the patient was allergic to gabexate mesylate, ulinastatin (100,000 U) was used to inhibit the activities of pancreatic enzymes. While ulinastatin was infused, the patient also developed allergic symptoms such as rash and itching. Ulinastatin was immediately stopped, and the allergic symptoms were significantly improved. Therefore, it was considered that the patient was also allergic to ulinastatin.

To sum up, the patient was allergic to ceftriaxone, gabexate mesylate, and ulinastatin. Upon inquiry, the patient stated that he had experienced redness, rash, and itching all over the body after taking painkillers (the painkiller is a compound preparation composed of acetaminophen, caffeine and aspirin) for cold, fever and headache. In addition, the patient reported that after taking ankahuangmin capsules (ankahuangmin capsule is a compound preparation composed of acetaminophen, caffeine, artificial bezoar, and chlorpheniramine maleate). The next day, he also exhibited similar symptoms. Moreover, these symptoms disappeared after stopping using these drugs. Considering that the patient was allergic to the painkillers and ankahuangmin capsules, the patient also showed that the skin would become red and swollen after using topical flurbiprofen cataplasms. In addition, the symptoms eased after stopping the drug. Therefore, it was considered that the patient was allergic to Flurbiprofen Cataplasms. On August 6, 2024, the patient was subjected to quantitative screening for autoimmune antibodies, inhalation allergens, and food allergens, and the results indicated no significant abnormalities (Table 2).

**Table 2:** Quantitative screening for inhalant and food allergens.

Number	Inspection item	Results	Indicate	level	Detection method
1	TIgE	964.10			Enzyme linked immunocapture assay (quantitative)
2	House dust mite	0.10	Negative	Level 0	Enzyme linked immunocapture assay (quantitative)
3	Dermatophagoides farinae	0.11	Negative	Level 0	Enzyme linked immunocapture assay (quantitative)
4	Cat epithelium	0.03	Negative	Level 0	Enzyme linked immunocapture assay (quantitative)
5	Dog epithelium	0.05	Negative	Level 0	Enzyme linked immunocapture assay (quantitative)
6	Peanut	0.05	Negative	Level 0	Enzyme linked immunocapture assay (quantitative)
7	Soybean	0.01	Negative	Level 0	Enzyme linked immunocapture assay (quantitative)
8	Milk	0.06	Negative	Level 0	Enzyme linked immunocapture assay (quantitative)
9	Crab	0.15	Negative	Level 0	Enzyme linked immunocapture assay (quantitative)
10	Shrimp	0.07	Negative	Level 0	Enzyme linked immunocapture assay (quantitative)
11	Eggs	0.13	Negative	Level 0	Enzyme linked immunocapture assay (quantitative)
12	Beef	0.11	Negative	Level 0	Enzyme linked immunocapture assay (quantitative)
13	Cod	0.03	Negative	Level 0	Enzyme linked immunocapture assay (quantitative)
14	Wheat flour	0.07	Negative	Level 0	Enzyme linked immunocapture assay (quantitative)
15	Mutton	0.01	Negative	Level 0	Enzyme linked immunocapture assay (quantitative)
16	House dust	0.07	Negative	Level 0	Enzyme linked immunocapture assay (quantitative)
17	Cockroaches	0.12	Negative	Level 0	Enzyme linked immunocapture assay (quantitative)
18	Alternaria	0.06	Negative	Level 0	Enzyme linked immunocapture assay (quantitative)
19	Willow	0.15	Negative	Level 0	Enzyme linked immunocapture assay (quantitative)
20	Common ragweed	0.05	Negative	Level 0	Enzyme linked immunocapture assay (quantitative)
21	Mugwort	0.02	Negative	Level 0	Enzyme linked immunocapture assay (quantitative)

Allergy level:

Level 0 (<0.35): negative.

Level 1 (0.35–0.70): low.

Level 2 (0.70–3.50): medium.

Level 3 (3.50–17.50): high.

Level 4 (17.50–50.0): very high.

Level 5 (50.0–100.0): pretty high.

Level 6 (>100.0): extremely high.

## 3 Discussion

In the United States and even most countries, acute pancreatitis exhibits a high incidence and is one of the primary causes of hospitalizations for gastrointestinal diseases (4). Recently, the incidence of acute pancreatitis in China is approximately (13–45)/100,000 people. The incidence rate in men is significantly higher than that in women. With the increase of age, the incidence and mortality rate are increasing, accordingly. Currently, the number of patients with acute pancreatitis admitted to the Department of Gastroenterology in our hospital is also on the rise year by year. China is confronting the risk of population aging, and thus the treatment of patients with pancreatitis is also a major challenge at present. There are many pathogeneses involved in acute pancreatitis. Among them, stone obstruction of the common bile duct, alcohol abuse, and hyperlipidemia are considered the most common causes of acute pancreatitis (5, 6). It has been reported that alcohol and smoking cessation can lower the risk of progression of pancreatitis and reduce its recurrence (7). Because the clinical manifestations of acute pancreatitis are complex, its clinical manifestations are easily confused with other acute abdominal diseases. In atypical cases, the diseases that clinicians need to distinguish and diagnose include acute cholecystitis and cholelithiasis (the abdominal pain of acute cholecystitis is milder than that of acute pancreatitis, the pain is located in the gallbladder area of the upper right abdomen, radiating to the right chest and the right shoulder, with the normal or slightly higher hematuria amylase; if accompanied by biliary stones, the abdominal pain is more severe, and is frequently accompanied by chills, high fever and jaundice). Perforation of gastric and duodenal ulcer (perforation of ulcer disease is sudden severe pain in the upper abdomen quickly spreading to the whole abdomen), coronary heart disease or myocardial infarction (in acute pancreatitis, abdominal pain may be reflexively radiated to the precordial area or produce a variety of electrocardiogram changes, which are often confused. However, patients suffering from coronary heart disease may have a history of coronary heart disease, a sense of pressure in the chest area, and insignificant abdominal signs, which must be carefully identified) and digestive tract tumors. Gabexate mesylate is a drug which can be mainly used for the basic treatment of acute pancreatitis in clinical practice (8). To relieve the symptoms of a patient with acute pancreatitis, the analgesic compound diclofenac sodium, the trypsin inhibitor gabexate mesylate, and the anti-infective drug ceftriaxone were administered. Allergic reactions including skin redness, rash, and itching were developed. Therefore, the above medical treatment may not be appropriate for this patient with acute pancreatitis. For this patient, during the treatment process, raw rhubarb was injected into gastric tube and enema to relieve constipation, mirabilite was applied externally to lower local exudation, and phloroglucinol was symptomatic to relieve pain. After a few days, the patient refused to continue treatment and stated that his condition had improved and asked to be discharged.

In our country, it is common for patients to be allergic to certain drugs, particularly antibiotics; however, allergies to multiple drugs are rare, and the underlying mechanisms are still unclear. So far, only one patient has been found to be allergic to multiple drugs in our hospital. The patient develops multiple drug allergies, the range of drug choices is narrowed, which brings great challenges to clinical treatment. Therefore, patients who have been confirmed to be allergic to multiple drugs should avoid contact with allergens and use allergic drugs. Once an allergic reaction occurs, the allergens should be immediately isolated and the allergic drugs should be withdrawn immediately. Emergency measures should be taken immediately for patients with severe allergies. Skin testing is an effective diagnostic method used for the diagnosis of allergic reactions. The only currently validated skin test for immediate allergies is penicillin, in which the antigenic determinant has been identified. However, skin tests for most other drugs have not yet been validated, while drug provocation tests can be employed to determine the authenticity of allergic reactions (3). In addition, drug provocation tests can determine true and false drug allergies, as well as formulate the best drug treatment plan based on the patient’s actual condition to improve medication efficacy and safety. Therefore, multiple departments should be combined to conduct drug provocation experiments. Meanwhile, multiple departments should consult to formulate appropriate treatment plans for these patients. In this study, after the patient developed multiple drug allergies, the doctor recommended that the patient should undergo drug provocation tests. However, after communicating with the patient and his family, they expressed their reluctance to perform drug provocation testing. Therefore, we respected the wishes of the patient and his family and did not conduct drug provocation testing.

## 4 Conclusion

To conclude, allergic reactions can endanger the patient’s life in some severe cases. Therefore, in clinical practice, it is essential to concentrate on allergic reactions caused by multiple drugs and take timely rescue measures after the occurrence of allergic reactions. Meanwhile, medication safety education should be provided to patients with multiple drug allergies and their families, aiming to avoid contact with allergens and drugs that can cause allergic reactions. For the patients with multiple drug allergies, personalized drug delivery is of great importance. Finally, clinical pharmacists should conduct full pharmaceutical supervision to ensure the safety and effectiveness of the treatment of such patients.

## Data Availability

The original contributions presented in the study are included in the article/supplementary material, further inquiries can be directed to the corresponding author.

## References

[1] Lankisch PG Apte M Banks PA. Acute pancreatitis. Lancet (London, England). (2015) 386:85–96. doi: 10.1016/S0140-6736(14)60649-8, PMID: 25616312

[2] Greenberg JA Hsu J Bawazeer M Marshall J Friedrich JO Nathens A. Clinical practice guideline: management of acute pancreatitis. Can J Surg. (2016) 59:128–40. doi: 10.1503/cjs.015015, PMID: 27007094 PMC4814287

[3] Blumenthal KG Saff RR Banerji A. Evaluation and management of a patient with multiple drug allergies. Allergy Asthma Proc. (2014) 35:197–203. doi: 10.2500/aap.2014.35.3739, PMID: 24801461

[4] Mederos MA Reber HA Girgis MD. Acute pancreatitis: a review. JAMA. (2021) 325:382–90. doi: 10.1001/jama.2020.20317, PMID: 33496779

[5] Spanier BW Dijkgraaf MG Bruno MJ. Epidemiology, aetiology and outcome of acute and chronic pancreatitis: an update. Best Pract Res Clin Gastroenterol. (2008) 22:45–63. doi: 10.1016/j.bpg.2007.10.007, PMID: 18206812

[6] Yang AL McNabb-Baltar J. Hypertriglyceridemia and acute pancreatitis. Pancreatology. (2020) 20:795–800. doi: 10.1016/j.pan.2020.06.005, PMID: 32571534

[7] Yadav D Lowenfels AB. The epidemiology of pancreatitis and pancreatic cancer. Gastroenterology. (2013) 144:1252–61. doi: 10.1053/j.gastro.2013.01.068, PMID: 23622135 PMC3662544

[8] Yoo YW Cha SW Kim A Na SY Lee YW Kim SH. The use of gabexate mesylate and ulinastatin for the prevention of post-endoscopic retrograde cholangiopancreatography pancreatitis. Gut Liver. (2012) 6:256–61. doi: 10.5009/gnl.2012.6.2.256, PMID: 22570757 PMC3343166

